# Horse bone marrow mesenchymal stem cells express embryo stem cell markers and show the ability for tenogenic differentiation by *in vitro *exposure to BMP-12

**DOI:** 10.1186/1471-2121-10-29

**Published:** 2009-04-22

**Authors:** Stefania Violini, Paola Ramelli, Laura F Pisani, Chiara Gorni, Paola Mariani

**Affiliations:** 1 Livestock Genomics Unit, Parco Tecnologico Padano, CERSA, Via Einstein, Loc Cascina Codazza, 26900, Lodi, Italy

## Abstract

**Background:**

Mesenchymal stem cells (MSCs) have been recently investigated for their potential use in regenerative medicine. MSCs, in particular, have great potential, as in various reports they have shown pluripotency for differentiating into many different cell types. However, the ability of MSCs to differentiate into tendon cells *in vitro *has not been fully investigated.

**Results:**

In this study, we show that equine bone marrow mesenchymal stem cells (BM-MSCs), defined by their expression of markers such as Oct4, Sox-2 and Nanog, have the capability to differentiate in tenocytes. These differentiated cells express tendon-related markers including tenomodulin and decorin. Moreover we show that the same BM-MSCs can differentiate in osteocytes, as confirmed by alkaline phosphatase and von Kossa staining.

**Conclusion:**

As MSCs represent an attractive tool for tendon tissue repair strategies, our data suggest that bone marrow should be considered the preferred MSC source for therapeutic approaches.

## Background

Over the past few years in veterinary medicine there has been an increased interest in understanding the biology of mesenchymal stem cells (MSCs). This interest comes from their potential clinical use especially in wound repair, tissue engineering and application in therapeutics fields, including regenerative surgery [1,2]. MSCs can be isolated directly from bone marrow aspirates [3], adipose tissue [4], umbilical cord [5] and various foetal tissues [6]. In the appropriate cell culture conditions they have the capacity to differentiate into several tissues [7], including bone [8], cartilage [9], muscle [10], adipose tissue and produce growth factors and cytokines promoting cell expansion and differentiation [11]. MSCs are an interesting model cell type to study differentiation mechanisms due to the relative ease of establishing *in vitro *cultures and their good proliferation [12]. Decades of experience have shown that tendons and ligaments regenerate and repair slowly and inefficiently *in vivo *after injury. Strain induced tendon injuries are a common consequence of athletic endeavour, in both horses and humans, often compromising a return to the previous level of activity. There are many similarities between the weight bearing tendons of the horse and human tendons, eg in matrix composition, and also in the nature of the injuries sustained. As tenocytes are highly differentiated cells, they have a limited potential for replication. Moreover, tenocytes are embedded in an extensive three-dimensional network of extracellular matrix components consisting mostly of type I, type III and type V proteoglycans, elastin and fibronectin [13-15].

The development of tendon tissue engineering for wound repair will depend on the identification and characterisation of appropriate sources of cells, as well as the development of new inert scaffolds for tissue regeneration [16]. The identification of an optimal cell source for tendon tissue engineering applications will require a rigorous characterisation of available sources for stem cells with regard to plasticity, propagation and control of differentiation. Under normal physiological conditions fully developed tendon is a poorly vascularised tissue with a low density of cells which exhibit low mitotic activities [17]. This could explain why tendon healing is slow and in most cases results in a mechanically inferior extracellular matrix [18]. Tissue engineering approaches have been investigated to improve tendon rupture healing by transplantation of *in vitro *cultured tenocytes, obtained from tendons, seeded in matrices [19-21]. These cells are highly specialised mesenchyme-derived cells, responsible for the synthesis and maintenance of a mechanically unique connective tissue, able to resist high tensile forces [22]. Nevertheless, a critical drawback of cellular transplantation approaches using *in vitro *cultured tenocytes is the loss of differentiated function that occurs during prolonged monolayer culture [23]. MSCs, under appropriate stimulation, represent an opportunity to produce *in vitro *cells of the required type. Bone morphogenetic proteins (BMPs) have been shown to induce differentiation of mesenchymal stem cells into osteogenic lineage [24]. The bone morphogenetic protein (BMP) family represents a subgroup of molecules within the transforming growth factor β (TGF-β) super family. BMPs were identified by their ability to promote ectopic cartilage and bone formation [25]. Among the BMPs isolated so far, BMP-2 and BMP-7 have been used to study tendon-bone healing in animal models [26]. Most importantly, it has been shown that gene transfer of BMP-12, a human homologue growth differentiation factor 7 (GDF7), is required to induce tenocyte differentiation process in MSCs [27]. Therefore, using BMP-12 to induce the development of MSCs into a sufficient number of tenocytes for the repair of a tendon defect was suggested [28]. Therefore adult stem cells, under specific stimulation represent a promising method to obtain *in vitro *tenocytes for tendon healing.

In this study we describe the *in vitro *growth of equine bone marrow MSCs. Cells were characterized using recognized molecular markers for "stemness" namely: Nanog, Oct4, Sox-2 and CD34. The undifferentiated cells were morphologically characterized and the presence of PCR products for the "stemness" markers assessed. The expression level of the latter was detected by QRT-PCR. Equine MSCs were induced to differentiate either into osteoblasts or into tenocytes by *in vitro *exposure to BMP-12. The results are relevant to future tissue engineering applications of this approach to human and equine MSCs in clinical practice.

## Results

### Cell morphology, growth characteristics and Oct4 expression by immunocytochemistry

**Figure 1:**
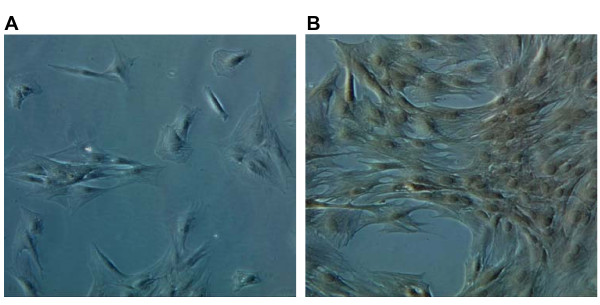
**BM-derived MSCs express stem cell marker protein Oct4**. Cells (5th passage) were fixed and incubated with antibodies directed against Oct4. Immunoreactivity was detected with avidin, biotinylated horseradish peroxydase and 3,3'-Diaminobenzidine (DAB) (**1B**). **1A**: negative control, incubated only with secondary antibody. Magnification 20×.

By day three after washing and removing the medium containing non-adherent cells, equine BM-MSCs appeared as isolated colonies of adherent elongated cells. Following further passages the equine BM-MSCs cells proliferated uniformly maintaining an homogeneous fibroblast-like morphology with a spindle shaped appearance and growing outward in a "swirling fibroblast-like" pattern. Nuclei were large and elliptical with the presence of a few nucleoli (data not shown). Between 3^rd ^and 10^th ^passage the BM-MSCs had a doubling time of 1,8 days. Immunocytochemical staining showed the expression of Oct4 in most of BM-derived stem cells (Fig. 1).

### Marker expression of BM-MS cells

**Figure 2:**
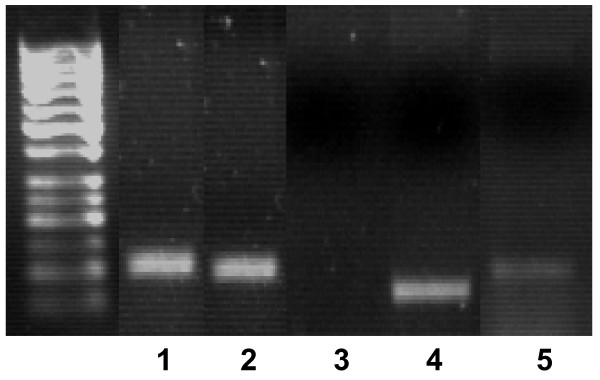
**Gene expression analysis of BM-MSCs**. The products from RT-PCR analysis of GAPDH (lane 1), Oct4 (lane 2), CD34 (lane 3), SOX-2 (lane 4) and Nanog (lane 5) mRNA expression at day 14 (passage 5) in BM-MSCs, showing the expression of mesenchymal stem cell-related markers and lack of expression of CD34.

The presence of Sox-2, Oct4 and Nanog amplicons in BM-MSCs was assessed: the expression of these genes is required for self-renewal and demonstrates multilineage differentiation potential [29,30]. The cells did not express the surface marker CD34, which is expressed in haematopoietic but not in MSCs (Fig. 2).

**Figure 3:**
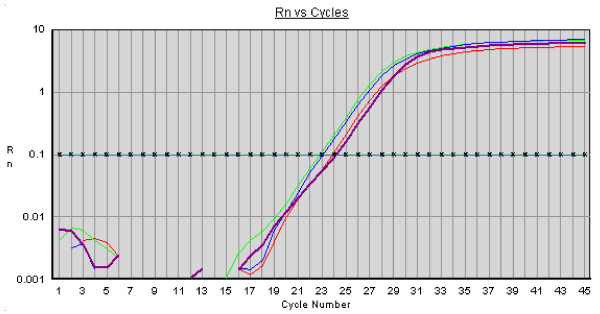
**QRT-PCR of BM-MSCs for the Nanog, Oct4, Sox2 and GAPDH genes**. Plot of QRT-PCR of Nanog (violet), Oct4 (green), Sox2 (blue) and GAPDH (red) on cDNA from BM-MSC cells. Cycle number is shown on the X-axis and emission intensity of a fluorescent reporter (Rn) is shown on the Y-axis. Fixed threshold is indicated as horizontal line.

The relative expression level of Sox-2, Oct4 and Nanog was assessed by QRT-PCR. Data showed these "stemness" markers to be expressed at comparable level with the constitutive expression of the housekeeping GAPDH gene (Fig. 3).

### Tenogenesis of BM-MSCs

**Figure 4:**
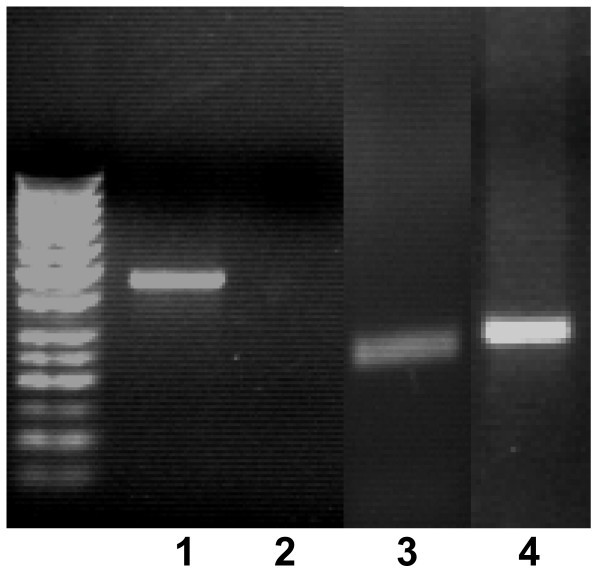
**Gene expression analysis of BM-MSC derived tenocytes**. Products from RT-PCR analysis of tenomodulin (lane 1), P19 lipocalin (lane 3), GAPDH (lane 3) and decorin (lane 4) mRNA expression following 14 days stimulation of BM-MSC cells with BMP-12, resulting in differentiation into tenocytes.

**Figure 5:**
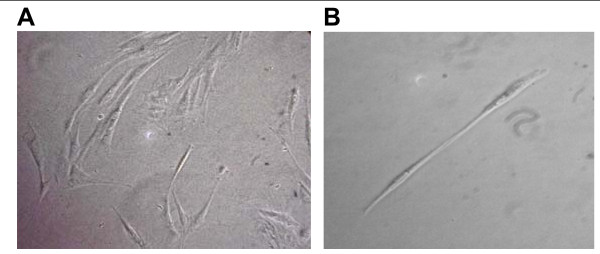
**Morphology of equine BM-MSC-derived tenocytes cultured in monolayer at day 20^th^**. The cells at 5^th ^passage in growth medium added with BMP-12 under phase contrast microscopy exhibited heterogeneous morphology with most cells fibroblast-like (A) and other elongated cells (B). Magnification 10×.

After tenogenesis induction cells were investigated for the expression of tenocytes markers and found to express tenomodulin, which is a tendon-specific gene [31,32] and decorin [33]. To rule out the possible unwanted differentiation into reproduction tissues, the expression of P19 lipocalin was checked (Fig. 4). Differentiated cells did not show P19 expression. This gene is not expressed in tenocytes and is expressed by horse uterine and endometrial tissues [34]. Compared to the untreated BM-MSCs, the differentiated cells showed a significant difference in morphology some being fibroblast-like and others having a more elongated tenocyte-like phenotype (Fig. 5).

### Osteogenic potential of BM-MSCs

**Figure 6:**
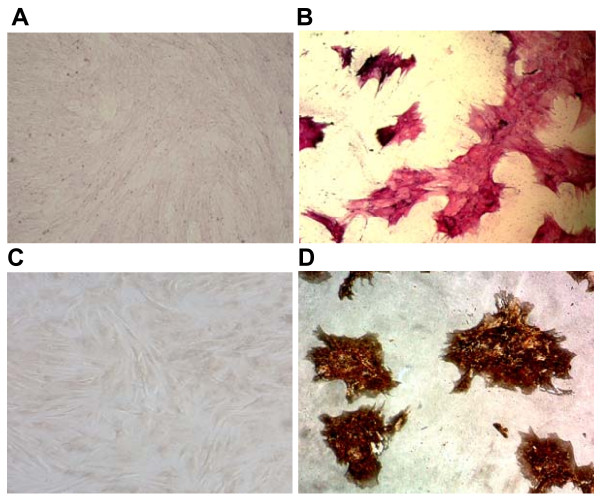
**BM-MSCs osteogenic induction: alkaline phosphatase (AP) and von Kossa staining**. After osteogenic induction cells showed a completely different morphology compared to untreated MSCs. (A, C): untreated control cells showing no staining for AP and von Kossa respectively (Magnification 10×). (B, D): AP and von Kossa positive staining in bone marrow-derived osteogenic cells. Magnification 20×.

Osteogenic differentiation was induced in the BM-MSCs by a medium containing β-glycerol phosphate, ascorbic acid and dexamethasone. The BM-MSCs formed calcium deposits identified by von Kossa staining (Fig. 6), suggesting the osteogenic potential of our MSCs culture. Moreover, cells stained positively for alkaline phosphatase, which is an indicator of osteogenic differentiation. Following osteogenic induction, the cells changed their morphology and showed more cubical and star-shaped morphology with spikely extensions and increased in size. Neither positive staining for alkaline phosphatase or mineralized matrix was observed in the cells cultured in regular growth medium.

## Discussion

Bone marrow mesenchymal stem cells (BM-MSCs) have been recently investigated as important source of undifferentiated cells in the field of regenerative medicine. As they can potentially differentiate into chondrocytes, adipocytes, osteocytes, myocardial and neural cells, they have become promising candidates as starting cells in tissue engineering studies aimed at improving wound healing and tendon tissue repair [35,36]. The technique of equine MSC isolation from bone marrow, *ex vivo *culture and expansion, has been previously reported [37]. Recently, Vidal described adipose-derive stem cell (ADSC) characteristics compared to BM-derived stem cells [38], mentioning their clinical relevance for tissue engineering applications in equine veterinary medicine, making adipose tissue a possible alternative source to BM.

Though ADSCs have been reported to display similar characteristics to BM-MSCs in humans in terms of gene expression profiles and phenotypes [39,40], their potential for both chondrogenic differentiation in 3D culture and proteoglycan synthesis and thus their quality in tissue engineering is controversial [40,41]. Winter and co-workers [40] found 90% reduced chondrogenic potential of ADSCs in micromass cultures compared to BM-MSCs and these observations were confirmed by Sakaguchi and co-workers [42], who analyzed various stem cell sources in chondrogenesis studies. Scientific literature has already shown that the methods to isolate BM-MSCs are faster than ADSCs and the yield in terms of purity is higher as in the latter case cells might be contaminated by mixed population of fibroblasts and adipocytes [43]. Moreover, Richardson and co-workers [43] reported MSCs isolation from fat is riskier than from bone marrow as there is more donor-site morbidity because of the surgery.

In general, tendon lesions in horses have been treated with BM-MSCs [35]. These cells have been largely used since they have been shown to be successful in repairing more specifically tendons and ligaments [36].

Adipose stem cell plasticity seems to be limited though compared to BM, as differentiation capabilities in tendon-like cells is still to be proven [44].

The focus of this study was to evaluate growth and differentiation of equine BM-MSCs. Adherent cells from bone marrow were found to actively proliferate *in vitro *and to maintain their morphological and growth characteristics for over 10 passages. Moreover, our results show that adherent equine BM-MSCs could proliferate rapidly in DMEM with FBS and EGF added with a doubling time of 1.8 day. This finding is comparable to previous studies that found an average cells doubling time of 1.4 days, with no significant difference in the doubling rate between foals and young horses BM-MSCs [38]. In our study cells grown in standard media showed an elongated fibroblast-like morphology, large cell size and capabilities of continuously dividing as has been observed in bone marrow stroma and tissue-specific MSC cells from other species [45,46]. RT-PCR analysis showed that Oct4, Nanog and Sox-2 genes were expressed by the stem cells. The following QRT-PCR showed these "stemness" markers were expressed at comparable level with the constitutive gene GAPDH. This data confirms that MSCs from bone marrow of adult animals also expresses embryonic stem cell markers.

Immunocytochemistry experiments confirmed that BM-MSCs expressed the embryo stem cell marker Oct4. These cells did not express the haematopoietic lineage marker CD34, thus confirming their characterisation as mesenchymal stem cells [46]. Interestingly, so far only embryonic stem cells had been found to express Oct4 and Nanog [47]. Recently, two papers reported the expression of Oct4 in equine umbilical cord cells [44,48]. In our study we show that also bone marrow-derived mesenchymal stem cells do express both Oct4 and Nanog. Literature data have considered embryo and adult stem cell markers as part of two different groups of markers. Our discovery that horse bone marrow MSCs express Oct4 and Nanog, together with a documented expression of SSEA-1 in murine MSCs by Anjos-Afonso [49], might indicate that the distinction between embryo and adult markers is not so strict. Having assessed in the present study that embryo stem cell markers are also expressed by adult stem cells, further investigations in the field are needed to identify which still unknown markers are unique and peculiar for embryo stem cells and to characterize embryological patterns in terms of differentiation capabilities. It should be noted that in our study the expression of equine cell markers was assessed by a PCR based panel of specifically designed oligonucleotide primers, instead of using antigen/antibodies based flow cytometry. This panel was used not only to assess the stemness of bone marrow-derived cells, but also to investigate their differentiation in tenocytes. This DNA marker-based panel represents a powerful tool in equine stem cells research, as many positive stem-cell marker antibodies, so far described in other species, show little or no cross-reactivity and thus cannot be used in the horse [45,46].

In this paper we show that horse BM-MSCs can be induced to differentiate in tenocytes. Following exposure to BMP-12 the BM-MSCs expressed two tendon-related markers, tenomodulin and decorin [31-33]. To rule out the possibility that BMP-12 had induced differentiation into other cell types, rather than tenocytes, P19 lipocalin expression was assayed. In the BM-MSCs no expression of this gene, which is known to be expressed in reproductive system tissues like endometrium and uterus [34] was assessed. The BM-MSCs also maintained their capability to differentiate into osteoblast lineage, which was confirmed by two different staining techniques to detect the presence of calcium deposits and positivity for alkaline phosphatase. In literature many reports have shown that MSCs have multilineage differentiation capabilities [7-10]. However, up to now there has been no report of tenocytes induction. Although the possibility of using transplanted mesenchymal stem cells for tissue repair has been suggested in rabbits [50], little it is known about capability of mesenchymal stem cells to differentiate into tissue-specific cell types *in vivo*. However, a combination of mechanical stimuli and proximity to tenocytes and tendon matrix are believed to be important as stimuli for differentiation into tendon cells, as shown by direct implantation of cells into the tendon. The transplantation of mesenchymal stem cells into injured tendons has been shown to promote tendon healing not only in laboratory animal models [50] but also in horses [51,52].

PLGA (poly(lactic-co-glycolic acid) fibres have been used as scaffolds in therapeutic approaches to tendon repair, owing to their biodegradability and biocompatibility [53]. However, 3D scaffolds have not shown any results in the case of tendon repair in horses. So far, many approaches have been investigated for improvements in tendon injury repair, but most are not completely understood and much further effort is necessary to develop the technology into a highly efficient treatment. The promise of functional tissue engineering to replace damaged organs or tissues has boosted research interest. At present, however, it is important to balance the understanding of our current limitations with a desire to progress the technology. The possibility to use MSCs that have been pre-differentiated into tendon cells for transplantation may represent a significant improvement over the use of undifferentiated cells.

For instance, there are some evidences of tumor induction by undifferentiated cells; more investigations in this matter, though, are needed [54].

## Conclusion

In summary, our study has confirmed, at molecular level through gene specific DNA markers and their expression patterns, that BM-MSCs possess the capability of differentiating into tenocytes. These data will be the basis of future efforts to standardize the isolation, expansion and transplantation of equine differentiated and undifferentiated stem cells in clinical practice.

## Methods

### Isolation of BM cells

Bone marrow (BM) samples were obtained aseptically from sternal aspirates of an 8-year-old Dutch Warmblood mare during surgery for hock arthroscopy under general anesthesia with the owner consensus at the Large Animal Hospital of the Faculty of Veterinary Medicine in Lodi. The harvest site was clean shaved, clipped and aseptically prepared.

The intersternebral spaces were easily identified by diagnostic ultrasonography and bone marrow needles (11 gauge, 10 cm) were used to aspirate marrow in two 20 ml syringes containing 30,000 units of sodium heparin each (Teofarma, Pavia, Italy). Immediately after collection, samples were stored on ice.

Preparation of MSC was achieved as follows. BM (30 ml) was layered over Hystopaque™ 1.077 (Sigma Aldrich, St. Louis, MO) and centrifuged for 20 min at 400 *g *and 4°C. The MSC-enriched cell population above the Hystopaque™ layer was aspirated and washed in calcium and magnesium-free Dulbecco's Phosphate Buffered Solution (PBS) by further centrifugation at 260 *g *for 5 minutes at 4°C. The cell pellets were resuspended in 10 ml Dulbecco Modified Earle's Medium (DMEM) supplemented with 10% fetal calf serum (FCS), penicillin (100 U/ml) and streptomycin (P/S) (100 μg/ml), non-essential amino acids (NEAA 1%), and seeded in 24-well plates (TPP, Trasadingen, Switzerland). All reagents were purchased from Euroclone (Milan, Italy).

Cells were checked for adhesion 3–4 days after plating and the medium was replaced with fresh medium containing Epidermal Growth factor (EGF) 50 ng/ml as specific growth factor (R&D System, Minneapolis, MN). After 2 weeks, when the cells were actively proliferating, they were sub-cultured every 3–4 days, counted and seeded in T25 flasks (4 × 10^5 ^cells/flask) or 24-well plates (2 × 10^5 ^cells/ml) and incubated at 37°C, 5% CO_2_. Photographs under AE30 Motic phase-contrast microscope were taken at passage 3.

### Proliferation study

To determine BM-MSCs doubling time, cells were plated at a density of 7,5 × 10^3 ^nucleated cells/well in a 6-well plate (Corning Inc, Corning, NY) and incubated in the growth medium described above. Cells were harvested daily sequentially from replicate cultures over seven consecutive days and cell number determined using a Burker chamber. Experiments were performed in triplicate. The mean number of cells was calculated and plotted on a common log scale against culture time to generate a growth curve. The mean doubling time was obtained by the formula [55]: PD = (lgNt-lgN_0_)/lg2 where N_0 _is the initial cell number; Nt is the cell harvest number at time t. Doubling times between day 1 and day 7 in culture were calculated.

### Immunocytochemistry

Cultured BM-derived stem cells were formalin fixed for 10 min, washed in PBS and permeabilized with 0.1% (v/v) Triton X-100 in PBS for 20 min. Cells were incubated with 1:50 specific goat polyclonal antibody raised against Oct4 (Santa Cruz Biotechnology, Germany) for 2 h at 37°C, followed by incubation for 2 h at room temperature with secondary biotin-conjugated donkey anti-goat IgG antibody (1:200) (Santa Cruz Biotechnology, Germany). Negative controls were incubated only with secondary antibody under the above condition. Excess secondary antibody was removed by repeated washing with PBS. Avidin, biotinylated horseradish peroxydase and 3,3'-Diaminobenzidine (DAB) were applied according to the manufacturer's instructions using Vectastain ABC kit (DBA Italia s.r.l., Italy). Images were obtained with a phase contrast microscope under a 20× magnification.

### "Stemness" Marker Analysis

#### RT-PCR reaction and conditions

Total RNA was isolated from BM-MSCs at 5^th ^passage using the RNeasy kit (Qiagen, Milan, Italy). Total RNA was dissolved in 30 μl of RNase-free water and was kept at -80°C until analysis. The RNA concentration was determined using a NanoDrop ND-1000 spectrophotometer (NanoDrop, Wilmington, DE). Total RNA (0.5 μg) was converted to cDNA with RevertAid H Minus M-MLV RT reverse transcriptase (Fermentas Int. Inc, Ontario). The obtained cDNA was amplified in 1× PCR buffer, with 0.5 μM sense and antisense primers, and 5 U Taq Gold polymerase (Applied Biosystem, Foster City, CA) using an Eppendorf Mastercycler Gradient (Eppendorf AG, Germany).

Gene-specific amplicons for the Nanog, Oct4, Sox2 and CD34 genes were obtained using primer pairs designed based on equine gene specific sequences where available, otherwise primers were designed using gene specific sequences from other species that are present in public databases (Table 1; primer sequences for the characterization protocol of equine stem cells are covered by patent applications owned by Fondazione Parco Tecnologico Padano).

**Table 1:** Polymerase chain reaction (PCR) primer sequences

**GAPDH**	F: CAACGAATTTGGCTACAGCA R: CTGTGAGGAGGGGAGATTCA
**CD34**	F: ATTACACGGAAAACGGTGGA R: AATTCGGTATCAGCCACCAC

**Oct4**	F: TCCCAGGACATCAAAGCTGCAGA R: GTCAAACTTACGTACCCTCTCGGGTCT

**NANOG**	F: TACCTCAGCCTCCAGCAGAT R: CATTGGTTTTTCTGCCACCT

**SOX-2**	F: TGGTTACCTCTTCCTCCCACT R: GGGCAGTGTGCCGTTAAT

**TENOMODULIN**	F: GATCTTCACTTCCCTACCAACG R: CTCATCCAGCATGGGGTC

**DECORIN**	F: GAATGAGATCACCAAGCTGC R: TGAGATGCGAATGTATGAGAGA

**LIPOCALIN**	F: TTCTTCATCCACAAGATCCAG R: AGTTGGGACACACATACCTCTT

The conditions for PCR amplification were as follows: 10 minutes at 95°C, followed by 35 cycles of 45 seconds at 95°C, 30 seconds at 58°C, 30 seconds at 72°C, followed by a final extension step of 5 minutes at 72°C. PCR products were resolved by electrophoresis on 2% agarose gels, visualized by ethidium bromide staining and photographed under ultraviolet light trans-illuminator (Bio-Rad, Hercules, CA).

#### Quantitative RT-PCR reaction and conditions

BM-MSCs cDNA was used as the template for QRT-PCR of Nanog, Oct4 and Sox2 genes. Analysis of GAPDH gene was added as reference for housekeeping gene expression level.

All reactions were carried out in a total volume of 10 μl, containing 0.5 μM of each primer, 5 μl of Sybrgreen master mix 2× (Applied Biosystems) and 5–10 ng cDNA. Each PCR reaction was carried out in triplicate and amplifications were performed using an ABI Gene Amplification 7900 Sequence Detection System (Applied Biosystems, Foster City, CA). A single optimized thermal protocol of 95°C for 10 minutes, 40 cycles of 95°C for 30 seconds, 61°C for 1 minute and a final extension at 72°C for 5 minutes was used for all primer pairs. Dissociation curve analysis was run to ensure the absence of non-specific product.

#### Data Analysis

The ΔΔC_T _method [56] was used to evaluate the relative expression levels among Nanog, Oct4 and Sox2 genes, using GAPDH as housekeeping gene.

### *In Vitro *Differentiation of BM-MSCs into tenocytes

At the 5^th ^passage when BM-MSCs were 70% confluent, cells were trypsinized, counted and plated at a density of 6 × 10^5 ^onto a T25 culture flask. To induce tenocyte differentiation, cell cultures were maintained for 14–21 days in growth medium supplemented with BMP-12 50 ng/ml (Tebu-Bio, Milan, Italy). The medium was changed every other day.

### Osteogenic differentiation

BM-MSCs were plated at density of 1 × 10^4 ^cells/well and cultured in a 6-well-plate. Induction medium consisted of DMEM-HG, 10% FCS, 1× P/S, 0.1 μmol/l dexamethasone, 0.025 mmol/l ascorbic acid-2-phosphate and 10 mmol/l β-glycerophosphate. All reagents were purchased from Sigma. Cells maintained in regular growth medium were used as negative control. After 2 weeks of induction, the cells were stained using the von Kossa procedure [57] to detect the presence of calcium deposition in osteocyte precursors. The positive and negative controls were also stained for alkaline phosphatase (Chemicon, Temecula, CA) according to the procedures described by the manufacturer. All the experiments were performed in triplicate.

## Authors' contributions

SV conceived of the study, carried out most of the experiments and drafted the manuscript. PR carried out some molecular studies. LFP participated in data collection. CG carried out some molecular studies and helped to draft the manuscript. PM conceived of the study, coordination and helped to draft manuscript. All the authors read and approved the final manuscript.
